# Sigma-2 ligands induce tumour cell death by multiple signalling pathways

**DOI:** 10.1038/bjc.2011.602

**Published:** 2012-01-17

**Authors:** C Zeng, J Rothfuss, J Zhang, W Chu, S Vangveravong, Z Tu, F Pan, K C Chang, R Hotchkiss, R H Mach

**Affiliations:** 1 Department of Radiology, Division of Radiological Sciences, Washington University School of Medicine, 510 S Kingshighway Boulevard, St Louis, MO 63110, USA; 2 Department of Anesthesiology, Washington University School of Medicine, 510 S Kingshighway Boulevard, St Louis, MO 63110, USA; 3 Department of Cell Biology and Physiology, Washington University School of Medicine, 510 S Kingshighway Boulevard, St Louis, MO 63110, USA; 4 Department of Biochemistry and Molecular Biophysics, Washington University School of Medicine, 510 S Kingshighway Boulevard, St Louis, MO 63110, USA

**Keywords:** sigma-2 receptors, apoptosis, autophagy, cell cycle, breast tumour cells

## Abstract

**Background:**

The sigma-2 receptor has been identified as a biomarker of proliferating cells in solid tumours. In the present study, we studied the mechanisms of sigma-2 ligand-induced cell death in the mouse breast cancer cell line EMT-6 and the human melanoma cell line MDA-MB-435.

**Methods:**

EMT-6 and MDA-MB-435 cells were treated with sigma-2 ligands. The modulation of multiple signaling pathways of cell death was evaluated.

**Results:**

Three sigma-2 ligands (WC-26, SV119 and RHM-138) induced DNA fragmentation, caspase-3 activation and PARP-1 cleavage. The caspase inhibitor Z-VAD-FMK partially blocked DNA fragmentation and cytotoxicity caused by these compounds. These data suggest that sigma-2 ligand-induced apoptosis and caspase activation are partially responsible for the cell death. WC-26 and siramesine induced formation of vacuoles in the cells. WC-26, SV119, RHM-138 and siramesine increased the synthesis and processing of microtubule-associated protein light chain 3, an autophagosome marker, and decreased the expression levels of the downstream effectors of mammalian target of rapamycin (mTOR), p70S6K and 4EBP1, suggesting that sigma-2 ligands induce autophagy, probably by inhibition of the mTOR pathway. All four sigma-2 ligands decreased the expression of cyclin D1 in a time-dependent manner. In addition, WC-26 and SV119 mainly decreased cyclin B1, E2 and phosphorylation of retinoblastoma protein (pRb); RHM-138 mainly decreased cyclin E2; and 10 *μ*M siramesine mainly decreased cyclin B1 and pRb. These data suggest that sigma-2 ligands also impair cell-cycle progression in multiple phases of the cell cycle.

**Conclusion:**

Sigma-2 ligands induce cell death by multiple signalling pathways.

Sigma receptors are a distinct class of receptors that are found in many tumours and normal tissues. Sigma receptors have been associated with many cellular and organ processes, including motor function, endocrine function, proliferation, immunoregulation and ion channel modulation (Walker *et al*, 1990; Hellewell *et al*, 1994; Megalizzi *et al*, 2010). Radioligand binding studies and biochemical analyses have shown that there are at least two types of sigma receptors, sigma-1 (∼25 kDa) and sigma-2 (∼21.5 kDa). The sigma-1 receptor has been cloned from many species (Hanner *et al*, 1996; Seth *et al*, 1997). Recently the progesterone receptor membrane component 1 (PGRMC1) protein complex was identified as the putative sigma-2 receptor-binding site (Xu *et al*, 2011). Sigma-2 receptors are expressed in high density in nearly all human and rodent tumour cell lines (Vilner *et al*, 1995). Sigma-2 receptor densities have been correlated with the grade of some human and bovine tumours (Bem *et al*, 1991; Colabufo *et al*, 2006; Roperto *et al*, 2010). The density of sigma-2 receptors in proliferating mouse mammary adenocarcinoma cells was found to be ∼10-fold higher than that in the corresponding quiescent tumour cells both *in vitro* and *in vivo* (Mach *et al*, 1997; Wheeler *et al*, 2000). These observations have led to the development of the sigma-2 ligands as molecular probes for diagnostic imaging of solid tumours (Wheeler *et al*, 2000; Mach *et al*, 2001a; Kawamura *et al*, 2003), and the identification of sigma-2 receptors as a potential target for treatment of cancer (John *et al*, 1998; Caveliers *et al*, 2001).

Apoptosis (programmed cell death) is a physiological process that is important for development, homeostasis and suppression of oncogenesis (Jacobson *et al*, 1997). Deregulated apoptosis has been implicated in many diseases, including ischemic stroke and cancer (Reed, 2002). It is well known that the caspase family has a central role in both the intrinsic and extrinsic pathways of apoptosis. Caspase-3, the ‘executioner’ caspase, is a key enzyme, which degrades proteins such as gelsolin and lamin, leading to apoptotic cell death (Reed, 2002). Sigma-2 ligands with different chemical structures have been reported to induce apoptosis in several tumour cell lines (Brent *et al*, 1996; Crawford and Bowen, 2002; Crawford *et al*, 2002, 2003; Barbieri *et al*, 2003; Ostenfeld *et al*, 2005; Cassano *et al*, 2009). Although two of these studies (Crawford and Bowen, 2002; Ostenfeld *et al*, 2005) have shown that prolonged exposure to sigma-2 ligands kills cancer cells by a caspase-independent apoptotic pathway, a complete understanding of how sigma-2-selective ligands induce cell death has not yet been established.

Autophagy is a lysosomal degradation pathway for cytoplasmic materials (Glick *et al*, 2010). At basal levels, autophagy maintains cellular homeostasis by removing misfolded or aggregated proteins, and by clearing damaged cellular organelles. Autophagy is strongly induced upon nutrient deprivation and enhances cell survival by degrading nonessential components of the cell to generate nutrients for vital biological processes. Autophagy begins with an isolation membrane, also known as a phagophore. The phagophore then expands to engulf cytoplasmic materials, forming a closed double-membrane autophagosome. Autophagosomes fuse with endosomal and/or lysosomal vesicles, promoting degradation of autophagosomal contents by lysosomal acid proteases. The degradation products can be re-used for cellular metabolism. By contrast, excessive autophagy can result in non-apoptotic cell death (Platini *et al*, 2010). Mammalian target of rapamycin (mTOR) promotes cell growth and inhibits autophagy (Janku *et al*, 2011). Mammalian target of rapamycin kinase forms two distinct multiprotein complexes called mTORC1 and mTORC2. Mammalian target of rapamycin-C1 activity results in phosphorylation of S6 ribosomal protein kinase (S6K), phosphorylation of eukaryotic initiation factor 4E-binding protein (4EBP1) and subsequent activation of the protein translational machinery in the cell. Mammalian target of rapamycin-C2 mediates Akt activation, which in turn stimulates and activates mTORC1. Because of its dual role in cell survival and cell death, both inhibition and activation of the autophagic lysosomal pathway are novel strategies for treating cancer (Platini *et al*, 2010). In fact, Ostenfeld *et al* (2008) have proposed combination therapy of siramesine, a sigma-2 ligand, with drugs that inhibit autophagy as a strategy for treating cancer.

The cell cycle can be described by four successive cellular phases: a phase of cell growth to prepare for DNA replication (G_1_), a phase of DNA synthesis and replication (S), and a phase of cell growth and active synthesis of factors (G_2_) required for mitosis (M) (Malumbres and Barbacid, 2009). Progression through the cell cycle is regulated by sequential waves of different cyclin/cyclin-dependent kinase (CDK) activities. Cyclins are synthesised and destroyed at specific time points during the cell cycle, thus regulating CDK kinase activities in a timely manner. Cyclin-dependent kinase–cyclin complexes directly involved in cell-cycle control include three interphase CDKs (CDK2, CDK4 and CDK6), a mitotic CDK (CDK1) and four classes of cyclins (cyclins A, B, D and E). Mitogenic signals first induced the expression of D-type cyclins (D1, D2 and D3). The D-type cyclins bind to and activate CDK4 and CDK6 during G_1_ phase, leading to phosphorylation of the retinoblastoma protein (Rb). Phosphorylation of Rb releases the E2F transcription factors, which can then activate genes essential for G_1_–S transition and S-phase, including E-type cyclins (Witzel *et al*, 2010). The E-type cyclins are required to activate Cdk2 for proper completion of the G_1_ phase, as well as for initiating DNA replication. Once cells enter S-phase, Cdk2–cyclin E complexes need to be silenced to avoid re-replication of DNA. The A-type cyclins accumulate during S-phase. Cyclin-dependent kinase-2–cyclin A complexes have been reported to phosphorylate numerous proteins that are required for proper completion and exit from S-phase. During G_2_, the A-type cyclins are degraded by ubiquitin-mediated proteolysis whereas the B-type cyclins are actively synthesised. As a consequence, Cdk1 binds to the B-type cyclins – an association believed to regulate several events during both the G_2_–M transition and progression through mitosis. Finally, inactivation of Cdk1–cyclin B complexes is required for proper exit from mitosis. This inactivation is accomplished by degradation of the B-type cyclins through the proteolytic pathway mediated by ubiquitination. Cell-cycle malfunction may cause cell death (Allan and Clarke, 2008; Clarke and Allan, 2009). Recent results indicate that regulation of apoptosis is directly linked with cell-cycle checkpoints by common components (Clarke and Allan, 2009). Few studies have investigated the effects of sigma-2 ligands on the cell cycle.

Our group has previously reported the synthesis and *in vitro* characterisation of a number of structurally diverse ligands with a high affinity for sigma-2 receptors (Mach *et al*, 2001b, 2003, 2004). By screening these sigma ligands for their cytotoxicity, we identified three potent sigma-2-selective ligands, WC-26, SV119 and RHM-138, that killed mouse breast cancer EMT-6 and human MDA-MB-435 melanoma tumour cells with EC_50_s in micromolar range after a 48-h exposure. The aim of the present study was to explore the mechanism by which these three sigma-2-selective ligands induce cell death. The data presented in this report indicate that treatment of EMT-6 and MDA-MB-435 cancer cells with these sigma-2 ligands induces multiple mechanisms of cell death, including apoptosis, autophagy and cell-cycle impairment.

## Materials and methods

### Cell culture conditions

EMT-6 mouse breast cancer cells were grown in DMEM containing 10% fetal bovine serum, 100 U ml^−1^ penicillin and 100 *μ*g ml^−1^ streptomycin. MDA-MB-435 human melanoma cells were grown in MEM containing 10% fetal bovine serum, 2 mM L-glutamine, 1 mM sodium pyruvate, 1% nonessential amino acids (Mediatech Inc., Manassas, VA, USA), 2% MEM vitamins (Invitrogen, Carlsbad, CA, USA), 100 U ml^−1^ penicillin and 100 *μ*g ml^−1^ streptomycin. Both cell lines were maintained at 37 °C in a humidified incubator under a 5% CO_2_/95% air atmosphere.

### MTS assay

The cytotoxicity of sigma-2 ligands was determined using the CellTiter96 Aqueous One Solution (Promega, Madison, WI, USA), which contains a tetrazolium compound (3-(4,5-dimethylthiazol-2-yl)-5-(3-carboxymethoxyphenyl)-2-(4-sulphophenyl)-2H-tetrazolium, inner salt; MTS).

### LDH assay

Lactate dehydrogenase (LDH) release assay was performed using the Cytotox 96 Non-Radioactive Cytotoxicity Assay (Promega) according to the manufacturer's protocol.

### Detection of intracellular caspase-3 activity

Activation of endogenous caspase-3 by sigma ligands was measured using the CellProbe HT caspase-3 whole-cell assay (Beckman Coulter, Fullerton, CA, USA) (see Supplementary Information for description of caspase-3 assay).

### Flow cytometry

Flow cytometric analysis was performed using a FACScan (Becton Dickinson, Fullerton, CA, USA) equipped with an air-cooled argon laser. TUNEL (terminal deoxynucleotidyl transferase dUTP nick-end labelling)-positive cells were detected with FITC–dUTP and quantified by flow cytometry using a TUNEL assay according to the manufacturer's protocol (Apo-direct Kit; BD Biosciences Pharmingen, San Diego, CA, USA). Briefly, the cells were detached from the culture dishes with trypsin, washed with phosphate-buffered saline (PBS) and then fixed in 1% paraformaldehyde. After washing with PBS twice, the cells were incubated with a DNA labelling solution containing terminal deoxynucleotidyl transferase and FITC–dUTP at 37 °C for 1 h. After washing with PBS, 7-amino-actinomycin D (7-AAD; BD Biosciences Pharmingen) was added to the cells to stain the nucleic acid at a concentration of 5 *μ*l (0.25 *μ*g) 7-AAD/test (1 × 10^6^ cells). The cells were then analysed by flow cytometry. Fluorescein isothiocyanate was excited at 488 nm and emission was collected with a 570-nm filter. 7-Amino-actinomycin D was excited at 488 nm and emission was collected using a 650-nm long-pass filter.

### Western blot analysis

EMT-6 cells (2 × 10^5^) or MDA-MB-435 cells (4 × 10^5^) were plated in 100-mm culture dishes 24 h prior to treatment with the sigma-2-selective ligands. For detection of caspase-3 activation, the cells were treated with WC-26 (40 *μ*M for EMT-6, 80 *μ*M for MDA-MB-435), SV119 (100 *μ*M) or RHM-138 (40 *μ*M). For detection of autophagy and cell-cycle impairment, MDA-MB-435 cells were treated with WC-26 (100 *μ*M), SV119 (100 *μ*M), RHM-138 (40 *μ*M) or siramesine (10 *μ*M). At various time points, cells were harvested and cell lysates were prepared using CHAPS buffer (50 mM Pipes/HCl (pH 6.5), 2 mM EDTA, 0.1% CHAPS (3-[(3-cholamidopropyl)-dimethylammonio]-1-propane sulphonate), 20 *μ*g ml^−1^ leupeptin, 10 *μ*g ml^−1^ pepstatin A and 10 *μ*g ml^−1^ aprotinin). Aliquots of protein (30 *μ*g) from each sample were analysed using standard immunoblotting procedures. Rabbit antibodies of caspase-3, PARP-1, LC3B, cyclin B1, cyclin D1, cyclin E2, pRb (Ser780), phospho-p70S6K (Thr389) and phospho-4EBP1 (Thr37/46), and a mouse antibody of cyclin A were purchased from Cell Signaling Technology (Danvers, MA, USA). All primary antibodies were used at a 1 : 1000 dilution. The secondary antibody was horseradish peroxidase (HRP)-conjugated goat anti-rabbit IgG or HRP-conjugated horse anti-mouse IgG (Cell Signaling Technology) at a 1 : 3000 or 1 : 10 000 dilution, respectively. The SuperSignal WestDura Extended Duration Substrate assay kit (Pierce Biotechnology Inc., Rockford, IL, USA) was used to detect the secondary antibody. For stripping western blots, the blot was incubated with stripping buffer (Pierce Biotechnology Inc.) for 15 min at room temperature.

### Transmission electron microscopy

MDA-MB-435 cells were plated in a 35-mm cell culture dish at a cell density of 3.2 × 10^5^ cells per dish for 24 h. The cells were then treated with 100 *μ*M WC-26 or 10 *μ*M siramesine for 0, 4, 8 and 16 h. The cells were quickly rinsed with PBS twice at room temperature and then fixed with 1 ml of 2.5% glutaraldehyde in 0.01 M Na cacodylate buffer at 4 °C until use. After rinsing with PBS, fixed cells were sequentially stained with osmium tetroxide and uranyl acetate, and then dehydrated and embedded in overturned gelatin capsules containing Polybed 812 resin (Polysciences, Warrington, PA, USA). The resin blocks were thin sectioned at 90–100 nm on a Reichert-Jung Ultracut microtome, post-stained in uranyl acetate and lead citrate, viewed on a Zeiss 902 Electron Microscope, and recorded with Kodak EM film.

### Statistical analysis

The results are expressed as the mean±s.d. based on three independent experiments performed in triplicate. Differences among groups were statistically analysed by two-tailed Student's *t*-test. A *P*-value of <0.05 was considered significant.

## Results

### Sigma-2 ligands induced cytotoxicity

**Figure 1:**
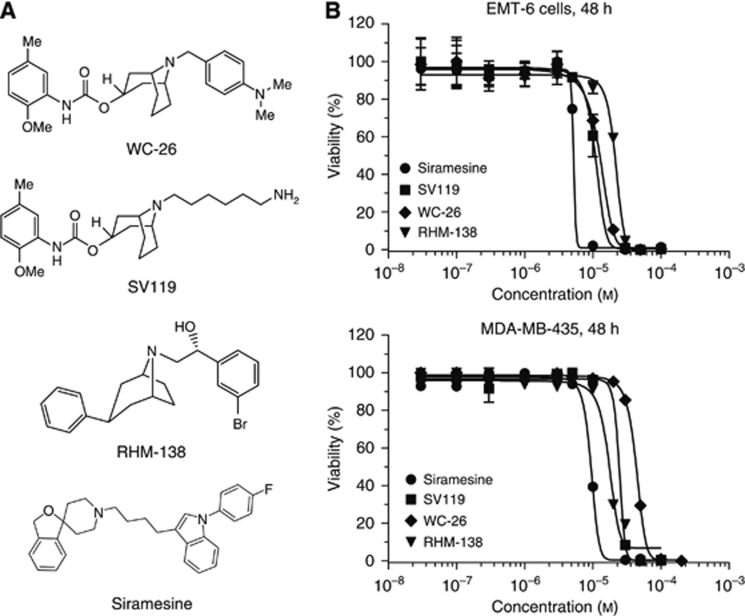
Sigma-2 ligands decreased viability in EMT-6 and MDA-MB-435 cells. (**A**) Chemical structures of the sigma-2 ligands. (**B**) EMT-6 or MDA-MB-435 cells were treated with increasing concentrations of the sigma-2 ligands WC-26, SV119, RHM-138 and siramesine for 48 h. Cell viability was determined by MTS assay. The bars represent the mean±s.e.m. of at least three independent experiments.

The chemical structures of the four sigma-2 ligands are shown in Figure 1A. Using the MTS assay, dose–response curves were generated after exposing EMT-6 and MDA-MB-435 cells to increasing concentrations of WC-26, SV119, RHM-138 and siramesine for 24 or 48 h. Cell kill increased with increasing dose of and exposure time to the four sigma-2 ligands (Figure 1B). All EC_50_s were in the micromolar range (Table 1).

### Sigma-2 ligands induced caspase-3 activation

**Figure 2:**
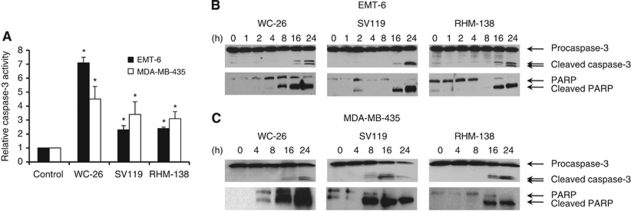
Sigma-2 ligands induced caspase-3 activation. (**A**) EMT-6 and MDA-MB-435 cells were treated for 24 h with the sigma-2 ligands at concentrations that resulted in the highest level of caspase-3 activation (40 *μ*M WC-26, 40 *μ*M SV119 or 40 *μ*M RHM-138 for EMT-6 cells; 80 *μ*M WC-26, 80 *μ*M SV119 or 50 *μ*M RHM-138 for MDA-MB-435 cells). Caspase-3 activation was determined by the CellProbe HT caspase-3 whole-cell assay. ^*^P<0.001 compared with untreated control. The bars indicate the mean±s.d. of the representative data of at least three independent experiments. (**B** and **C**) Sigma-2 ligands induced procaspase-3 and PARP-1 cleavage as shown by western blot analysis in EMT-6 cells (**B**) and MDA-MB-435 cells (**C**). EMT-6 cells were treated for 0–24 h prior to assay with WC-26 (40 *μ*M), SV119 (100 *μ*M) or RHM-138 (40 *μ*M). MDA-MB-435 cells were treated for 0–24 h with WC-26 (80 *μ*M), SV119 (100 *μ*M) or RHM-138 (40 *μ*M).

To assess if caspase-mediated apoptosis was involved in the cytotoxic pathway, caspase-3 activity was measured by a whole-cell assay described under Materials and Methods. The EMT-6 cells were treated with 40 *μ*M WC-26, 40 *μ*M SV119 or 40 *μ*M RHM-138 for 24 h. The data showed that caspase-3 activation in treated cells increased by 7-, 2.5- and 2.5-fold, respectively, over activation measured in untreated control cells (Figure 2A). MDA-MB-435 cells were also treated for 24 h with 80 *μ*M WC-26, 80 *μ*M SV119 or 50 *μ*M RHM-138, and caspase-3 activities were shown to increase by 4.5-, 3.5- and 3-fold, respectively (Figure 2A).

Caspase-3 activation was also demonstrated by western blot analysis. Activation of caspase-3 requires proteolytic processing of inactive procaspase-3 (35 kDa) into inactive 19-kDa and active 17-kDa, and 12-kDa caspase-3, fragments. EMT-6 (Figure 2B) and MDA-MB-435 cells (Figure 2C) were treated for 0–24 h prior to the assay. All three sigma-2 ligands induced procaspase-3 cleavage in both EMT-6 and MDA-MB-435 cells after an 8- to 24-h treatment. Poly(ADP-ribose) polymerase-1 (PARP-1), the 116-kDa poly(ADP-ribose) polymerase, is one of the main cleavage targets of caspase-3 *in vivo*. The western blot analyses demonstrated that all three sigma-2 ligands induced PARP-1 cleavage in both cell lines (Figure 2B and C). Collectively, these data indicate that our three sigma-2 ligands induced cell death through an apoptotic pathway.

**Figure 3:**
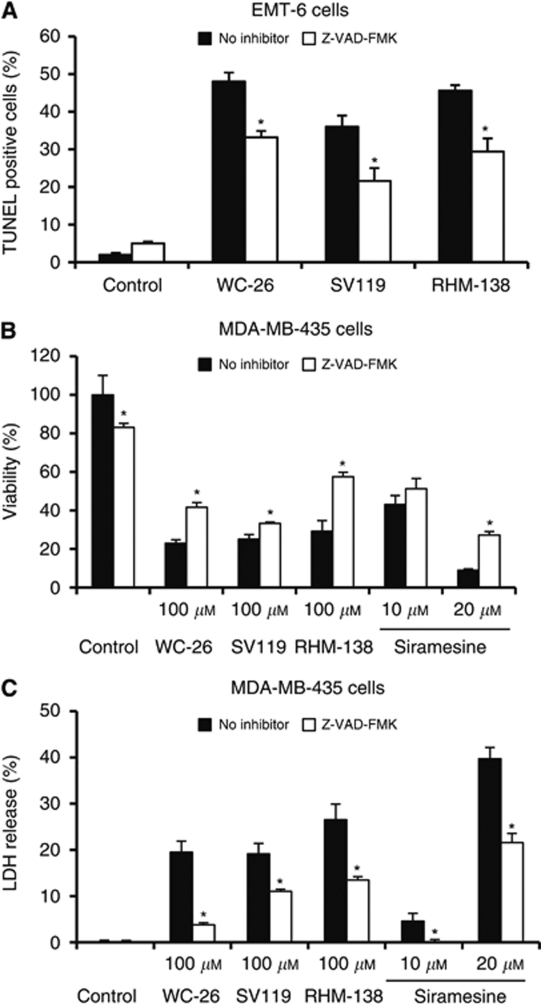
The broad-spectrum caspase inhibitor, Z-VAD-FMK, partially blocked DNA fragmentation, viability and cytotoxicity induced by sigma-2 ligands. (**A**) EMT-6 cells were pre-treated for 1 h with Z-VAD-FMK (100 *μ*M), and then treated with WC-26 (40 *μ*M) for 48 h, SV119 (100 *μ*M) for 16 h or RHM-138 (40 *μ*M) for 16 h. The percentage of TUNEL-positive cells was analysed by flow cytometry (^*^P⩽0.001 compared with the no inhibitor control). (**B** and **C**) MDA-MB-435 cells were pre-treated for 1 h with Z-VAD-FMK (100 *μ*M), and then treated with WC-26 (100 *μ*M), SV119 (100 *μ*M), RHM-138 (100 *μ*M) or siramesine (10 or 20 *μ*M) for 18 h. Viability of the cells was measured by MTS assay (**B**). Cytotoxicity to the cells was measured by LDH assay. (^*^P<0.05 compared with the no inhibitor control). The bars represent the mean±s.d. of the representative data of at least three independent experiments.

To determine whether caspases are responsible for the sigma-2 ligand-induced apoptosis and cell viability, the broad-spectrum caspase inhibitor, Z-VAD-FMK, was added to the culture medium 1 h before sigma-2 ligands. The effect of this inhibitor on sigma-2 ligand-induced DNA fragmentation was determined by TUNEL staining. The effect of the inhibitor on cell viability following treatment with sigma-2 ligands was determined by MTS assay and LDH assay. The results showed that DNA fragmentation induced by each of the three sigma-2 ligands was partially blocked by Z-VAD-FMK (100 *μ*M) in EMT-6 cells (Figure 3A). The decreased cell viability by the sigma-2 ligands was partially rescued by Z-VAD-FMK (Figure 3B and C). These data suggest that caspase activation, to a certain extent, has a role in sigma-2 ligand-induced cell death. It is worth noting that Z-VAD-FMK partially blocked cell death induced by 20 *μ*M siramesine but not by 10 *μ*M siramesine (Figure 3B), suggesting that the role of caspase in sigma-2 ligand-induced cell death is concentration-dependent.

### Sigma-2 ligands induced autophagy

**Figure 4:**
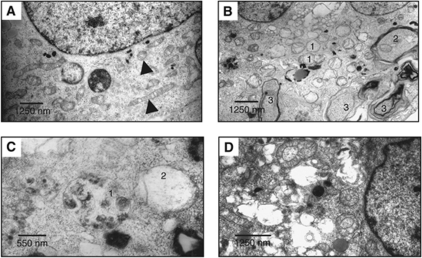
WC-26 and siramesine induced the formation of autophagosomes in MDA-MB-435 cells studied by transmission electron microscopy. (**A**) Cells without any treatment showed normal mitochondria ultrastructure (arrows). (**B**) Treatment with 100 *μ*M WC-26 for 4 h induced mitochondrial swelling (indicated by 1), multilayer membrane compartments containing mitochondria and other cytoplasm contents (indicated by 2), and various kinds of multilayer membrane structures (indicated by 3). (**C**) Treatment with 100 *μ*M WC-26 for 8 h induced autophagic vacuoles (indicated by 1) and mitochondrial swelling (indicated by 2). (**D**) Treatment with 10 *μ*M siramesine for 16 h induced the formation of various vacuoles.

**Figure 5:**
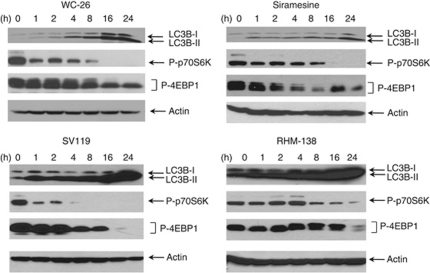
Sigma-2 ligands induced autophagy in MDA-MB-435 cells. Cells were treated with WC-26 (100 *μ*M), SV119 (100 *μ*M), RHM-138 (40 *μ*M) or siramesine (10 *μ*M) for 0–24 h. Autophagosome markers, LC3B and the downstream effectors of mTOR, p70S6K and 4EBP1, were analysed by western blotting.

Excess levels of autophagy may be responsible for cell death (Platini *et al*, 2010). We therefore examined whether autophagy was involved in sigma-2 ligand-induced cell death. We first used transmission electron microscopy to examine the ultrastructure of MDA-MB-435 cells after treatment with 100 *μ*M WC-26 or 10 *μ*M siramesine. Compared with control cells (Figure 4A), 4-h treatment with 100 *μ*M WC-26, the earliest time point examined, induced mitochondrial swelling (Figure 4B, structure indicated by 1) and multilayer membrane structures (Figure 4B, structures indicated by 2 and 3). Typical autophagy compartments, which have double membranes and partially degraded cytoplasmic material, were occasionally observed (Figure 4C, structure 1). Siramesine at 10 *μ*M also induced autophagy-like vacuoles (Figure 4D). These ultrastructures suggest that autophagy may be induced by sigma-2 ligands. We further used western blot analysis to examine the processing of microtubule-associated protein light chain 3 (LC3), an autophagosome marker. Light chain 3 is expressed in most cell types as a full-length cytosolic protein, which exists in three isoforms (LC3A, LC3B and LC3C) and is proteolytically cleaved upon induction of autophagy, generating LC3-I. The carboxy-terminal glycine of LC3-I then conjugates to phosphatidylethanolamine to generate processed LC3-II. Light chain 3-II is found on autophagosomes, where it has a role in both membrane fusion and selection of cargo for degradation (Barth *et al*, 2010). Western blot analysis demonstrated that LC3B-II was induced by all four sigma-2 ligands (100 *μ*M WC-26, 100 *μ*M SV119, 40 *μ*M RHM-138 and 10 *μ*M siramesine) over a 24-h period (Figure 5). We also tested whether the mTOR signalling pathway is involved in sigma-2 ligand-induced autophagy. Western blot analysis showed that phosphorylation of p70S60 kinase and 4EBP-1, both of which are downstream effectors of mTORC1, were decreased by all four sigma-2 ligands, suggesting that the mTOR pathway is inhibited by sigma-2 ligands, which may in turn trigger autophagy (Figure 5).

### Sigma-2 ligands impaired the cell cycle in MDA-MB-435 cells

**Figure 6:**
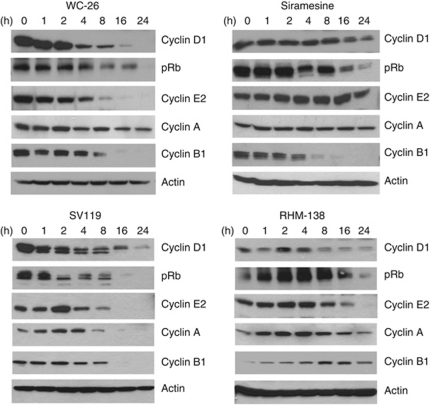
Sigma-2 ligands impaired cell-cycle progression in MDA-MB-435 cells. Cells were treated with WC-26 (100 *μ*M), SV119 (100 *μ*M), RHM-138 (40 *μ*M) or siramesine (10 *μ*M) for 0–24 h. The expression levels of cyclin D1, pRb, cyclin E2, cyclin A and cyclin B1were analysed by western blotting.

Deregulation of cell-cycle progression may cause cell death (Allan and Clarke, 2008; Clarke and Allan, 2009). Therefore, we examined the effects of sigma-2 ligands on the expression levels of all four major classes of cyclins (cyclin D1, E2, A and B1) and on the phosphorylation levels of Rb by western blot analysis. MDA-MB-435 cells were treated with 100 *μ*M WC-26, 100 *μ*M SV119, 40 *μ*M RHM-138 or 10 *μ*M siramesine for 0, 1, 2, 4, 8, 16 and 24 h. The data showed that all four sigma-2 ligands decreased the expression levels of cyclin D1 in a time-dependent manner (Figure 6). In addition, the data showed that 100 *μ*M WC-26 or 100 *μ*M SV119 mainly decreased cyclin B1, E2 and phospho-Rb (pRb); 40 *μ*M RHM-138 mainly decreased cyclin E2; and 10 *μ*M siramesine mainly decreased cyclin B1 and pRb. It is well known that D-type cyclins are responsible for progression through G_1_ phase; E-type cyclins for completion of G_1_ phase and entry into S-phase; A-type cyclins for driving the transition from S-phase to M-phase (G_2_ phase); and B-type cyclins for cell progression through M-phase (Malumbres and Barbacid, 2009). Our data suggest that all four sigma-2 ligands may block G_1_-phase progression by decreasing cyclin D1 expression. In addition, WC-26 and SV119 may block cell entry from G_1_ to S-phase by decreasing cyclin E2, and block mitosis by decreasing cyclin B1; RHM-138 may block cell entry from G_1_ to S phase by decreasing cyclin E2; and siramesine may block mitosis by decreasing cyclin B1. Collectively, our data suggest that all four sigma-2 ligands may impair the cell cycle in multiple phases (G_1_, S, G_2_ and M), which may lead to apoptosis either directly or indirectly, as discussed below.

## Discussion

We studied the cell death mechanisms of three sigma-2 receptor ligands developed in our group and compared the results with the known sigma-2 agonist, siramesine. All four sigma-2 ligands induced cell death in EMT-6 and MDA-MB-435 cells. Our results indicated that (1) the apoptotic pathway is, in part, responsible for cell death; (2) the sigma-2 ligands induced autophagy; and (3) the sigma-2 ligands impaired cell-cycle progression.

We first examined whether the sigma-2 ligands induced cell death through activating apoptosis. All three ligands induced Annexin-V-positive cells (Supplementary Figure 1), DNA fragmentation, caspase-3 activation and PARP-1 cleavage. In addition, at least one sigma-2 ligand, RHM-138, activated caspase-8 and 9 (Supplementary Figure 2). These results suggest that sigma-2 ligands induced apoptosis possibly through both intrinsic and extrinsic pathways. This conclusion is consistent with that reported from other laboratories (Crawford and Bowen, 2002; Ostenfeld *et al*, 2005). Our data also showed that the broad-spectrum caspase inhibitor, Z-VAD-FMK, partially inhibited DNA fragmentation and cytotoxicity of the sigma-2 ligands, suggesting that caspases are, in part, responsible for sigma-2 ligand (WC-26, SV119 and RHM-138)-induced cell death in EMT-6 and MDA-MB-435 cells. In previous studies, Crawford *et al* showed that the caspase inhibitors had no effect on sigma-2 ligand (CB-64D and CB-184) cytotoxicity or Annexin-V binding in human breast MCF-7 cells (Crawford and Bowen, 2002). Ostenfeld *et al* (2005) reported that caspase inhibitors failed to protect cells against siramesine-induced death in murine fibrosarcoma cells (WEHI-S) and MCF-7 cells. It appears that whether caspases are involved in sigma-2-induced cell death depends on the structure of the ligand, ligand concentration and tumour cell type.

We examined whether sigma-2 ligands induced autophagy. Electron microscopic data showed that the sigma-2 ligands WC-26 and siramesine induced mitochondrial swelling and formation of multilayer membrane vacuoles (Figure 4A–D). The sigma-2 ligands also increased the expression of the autophagosome marker LC3B, and decreased the downstream effectors of mTOR, p70S6K and 4EBP1 (Figure 5). These results suggest that sigma-2 ligands induce autophagy most likely by inhibiting the mTOR pathway. These data are consistent with the previous report that siramesine induced autophagosome formation in MCF-7 cells (Ostenfeld *et al*, 2008). Autophagy has a dual role in cell survival and cell death. On one hand, autophagy is generally thought of as a survival mechanism through removal misfolded proteins and damaged organelles. On the other hand, excess levels of autophagy may lead to cell death (Platini *et al*, 2010). Ostenfeld *et al* (2008) showed that 3-methyladenine, an autophagy inhibitor, increased cytotoxicity induced by siramesine. They also showed that inhibition of autophagy by RNA interference-based depletion of the autophagy protein beclin 1 increased cell toxicity of siramesine. These data suggest that siramesine-induced autophagy is cytoprotective in MCF-7 cells and NIH3T3 cells. Studies of a variety of experimental systems indicate that whether autophagy is cytoprotective or cytotoxic is likely to be context- and cell type-dependent (Platini *et al*, 2010). The role of our sigma-2 ligands, WC-26, SV119 and RHM-138, in autophagy in MDA-MB-435 cells needs to be further studied.

In the current work, we studied the effects of sigma-2 ligands on cell-cycle progression by examining the protein levels of the four major classes of cyclins. Our data showed that sigma-2 ligands altered cyclin protein expression levels and the phosphorylation levels of Rb (Figure 6). These data suggest that sigma-2 ligands may induce cell death by impairing cell-cycle progression. Regulation of cyclin levels has been extensively studied. Cyclin D1 protein is subject to transcriptional regulation and ubiquitin-mediated proteolysis (Witzel *et al*, 2010). Unlike other cyclins, cyclin D1 is strongly dependent on extracellular mitogenic signals. For example, cyclin D1 is induced by the Ras-signalling pathway (Malumbres and Barbacid, 2001). It is possible that sigma-2 ligands decrease cyclin D1 by inhibiting the mitogenic signalling pathways or/and by increasing ubiquitin-dependent degradation processes. The Rb represses transcription by binding to transcription factors such as E2F-family members. D-type cyclins in complexes with CDK4 and/or CDK6 result in phosphorylation of Rb (Malumbres and Barbacid, 2005). This liberates E2F transcription factors and promotes the subsequent transcription of many important proteins such as cyclin E. In the present study, we show that the phosphorylation levels of Rb are decreased by WC-26, SV119 and siramesine. This could result from cyclin D1 reduction upon treatment with these ligands. The E-type cyclins are regulated transcriptionally by the phosphorylation status of Rb as well as by ubiquitin-dependent degradation mechanisms (Musgrove, 2006; Caldon and Musgrove, 2010). Our data show that WC-26 and SV119 decrease cyclin E2. This is probably due to reduction of phospho-Rb. By contrast, siramesine does not reduce cyclin E2. One possible reason is that siramesine decreases cyclin E2 by reducing phospho-Rb, but at the same time increases cyclin E2 by inhibiting its degradation. The net result is no change of cyclin E2 expression. A- and B-type cyclins are also regulated at the transcriptional level and by ubiquitin-mediated proteolysis (Miyazaki and Arai, 2007). The current study shows that WC-26, SV119 and siramesine caused a dramatic decrease in cyclin B1 expression. This could result from decreasing the transcription and/or increasing the ubiquitin-dependent degradation processes. Taking these data together, the sigma-2 ligands decrease multiple cyclin protein levels. Decreasing cyclin levels has been proposed as a therapeutic strategy for cancer treatment (Freemantle *et al*, 2007). Our findings suggest that the sigma-2 ligands may serve as promising antitumour drugs by disrupting cell-cycle progression.

Sigma-2 ligands induce apoptosis, autophagy and cell-cycle impairment. These pathways are inter-related. Autophagy and cell-cycle arrest are known adaptive responses of cells to toxic insults (Clarke and Allan, 2009; Wyllie, 2010). Cells can remove the misfolded proteins and damaged organelles by autophagy. Cells can also activate cell-cycle checkpoints, which provide an opportunity to repair the damage. It is possible that upon sigma-2 ligand treatment, cells initiate autophagy and cell-cycle arrest to protect themselves from the toxic effects of the ligands. After these mechanisms fail to rescue the cells, they switch to facilitate cell death. Understanding the turning points where adaptive responses become contributors to toxicity is important for designing effective cancer therapeutic interventions. The PI3K/Akt/mTOR pathway is well known as one of the key molecular switches between apoptosis and autophagy (Moretti *et al*, 2007; Wyllie, 2010; Li *et al*, 2011). Our data suggest that sigma-2 ligands increase autophagy and apoptosis by suppressing the mTOR pathway. In addition, recent studies have revealed that the cell-cycle components can be directly involved in apoptosis. It is reported that CDK1–cyclin B1 is a crucial regulator of not only mitosis but also apoptosis (Clarke and Allan, 2009). Cyclin-dependent kinase-1–cyclin B1 phosphorylates the inhibitory site on caspase-9 (Thr125) during mitosis (Allan and Clarke, 2007). Phosphorylation of this site restrains caspase-9 activation and apoptosis. It is possible that the decrease in cyclin B1 upon treatment of the sigma-2 ligands reduces the phosphorylation level of caspase-9 at the inhibitory site and thus activates caspase-9 and apoptosis.

Previously we have shown that the sigma-2 fluorescent ligands rapidly internalise into cells and localise in the mitochondria, lysosomes, endoplasmic reticulum and plasma membranes (Zeng *et al*, 2007, 2011). It is possible that sigma-2 ligands trigger multiple signalling pathways by binding to sigma-2 receptors located in multiple cytoplasmic organelles and plasma membranes. For example, sigma-2 ligands may bind to mitochondria and damage their structure and function, as shown by mitochondrial swelling (Figure 4B and C), and trigger caspase-3-dependent or -independent apoptosis (Crawford and Bowen, 2002; Ostenfeld *et al*, 2005). Sigma-2 ligands may bind to lysosomes, and cause lysosomal leakage and cell death as demonstrated previously by Ostenfeld *et al* (2005). The damage to cytoplasmic organelles by sigma-2 ligands may trigger autophagy, which may have either a survival or a detrimental role depending on the structure of the drugs and the tumour cell type.

Positron emission tomography imaging in rodent (Mach *et al*, 2001a; Kawamura *et al*, 2003) and human studies (unpublished data) has demonstrated that sigma-2 ligands selectively target tumours *in vivo*. Sigma-2 receptor ligands have shown efficacy in various tumour cells using cell culture and animal models (Ostenfeld *et al*, 2005; Kashiwagi *et al*, 2007, 2009) by multiple signalling pathways. These characteristics make sigma-2 ligands promising chemotherapeutic drugs for treating a variety of tumours.

## Figures and Tables

**Table 1:** Cytotoxicity of sigma-2 ligands

	**EMT-6**	**EMT-6**	**MDA-MB-435**	**MDA-MB-435**
**Compound**	**EC_50_ (*μ*M, 24 h)**	**EC_50_ (*μ*M, 48 h)**	**EC_50_ (*μ*M, 24 h)**	**EC_50_ (*μ*M, 48 h)**
WC-26	42.5±3.5	12.3±1.6	49.7±2.5	42.6±2.3
SV119	16.0±1.4	11.4±1.7	36.7±3.33	18.5±1.9
RHM-138	32.5±3.5	19.5±2.0	26.7±4.25	17.8±1.6
Siramesine		5.3±1.0		9.3±0.9
