# Supplementary material for: Sigma-2 ligands induce tumour cell death by multiple signalling pathways
Source: Br J Cancer. 2012 Jan 17;106(4):693–701. doi: 10.1038/bjc.2011.602 (PMC3322954; doi:10.1038/bjc.2011.602)
Supplement: Supplementary Figures 1 and 2 [file bjc2011602x1.ppt]

## Slide 1
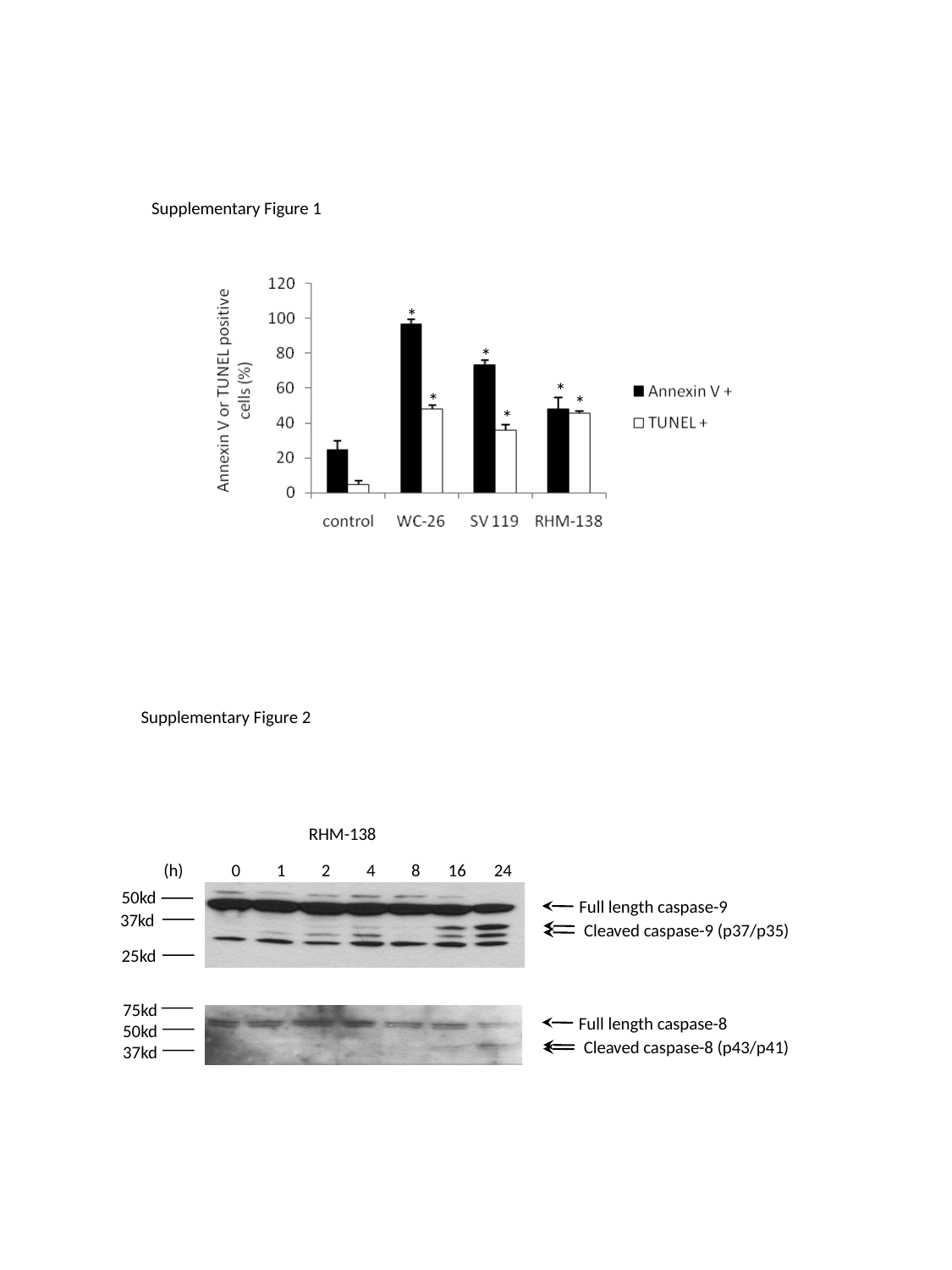

Supplementary Figure 1
*
*
*
*
*
*
Supplementary Figure 2
RHM-138
 (h) 0 1 2 4 8 16 24
50kd
Full length caspase-9
37kd
 Cleaved caspase-9 (p37/p35)
25kd
75kd
Full length caspase-8
50kd
 Cleaved caspase-8 (p43/p41)
37kd
