# Supplementary material for: Sigma-2 ligands induce tumour cell death by multiple signalling pathways
Source: Br J Cancer. 2012 Jan 17;106(4):693–701. doi: 10.1038/bjc.2011.602 (PMC3322954; doi:10.1038/bjc.2011.602)
Supplement: Supplementary Information [file bjc2011602x2.doc]

**SUPPLEMENTARY INFORMATION**

**MATERIALS AND METHODS**

**MTS assay**

The cytotoxicity of sigma-2 ligands was determined using the CellTiter96 Aqueous One Solution Assay (Promega, Madison, WI). Briefly, EMT-6 or MDA-MB-435 cells were seeded in a 96-well plate at a density of 2000 cells/well on the day prior to treatment with sigma-2 selective ligands. After a 24 or 48 hour treatment, the CellTiter 96 AQueous One Solution Reagent was added to each well, and the plate incubated for 2 hours at 37°C. The plate was then read at 490 nm in a Victor^3^ plate reader (**PerkinElmer Life and Analytical Sciences**, Shelton, CT). The EC_50_ value, defined as the concentration of the sigma ligand required to inhibit cell viability by 50% relative to untreated cells, was determined from the dose response curve for each cell line.

**LDH assay**

The lactate dehydrogenase (LDH) release assay was performed using the Cytotox 96® Non-Radioactive Cytotoxicity Assay (Promega, Madison, WI) according to the manufacturer’s protocol. Briefly, MDA-MB-435 cells were seeded at 2 x 10^3^ cells/well in 96-well plates on the day prior to treatment with the sigma-2 ligands. After a 24 hour treatment with the various sigma ligands, plates were centrifuged at 250 x *g* for 5 minutes. 50 µl of supernatant was removed and transferred to a new plate. 50 µl of the reconstituted substrate mix was then added to each well and the plate was allowed to incubate at room temperature for 30 minutes, protected from light. After 30 minutes, 50 µl of stop solution was added to each well and the plate was read at 490 nm in a Victor^3^ plate reader (PerkinElmer Life and Analytical Sciences, Shelton, CT).

**Detection of intracellular caspase-3 activity**

The activation of endogenous caspase-3 by the sigma ligands was measured using the CellProbe HT caspase-3 whole cell assay (Beckman Coulter, Fullerton, CA). Briefly, the cell permeable, nonfluorescent bisamide substrate, Z-DEVD-R110, is added to the intact cells to detect apoptosis. Upon specific cleavage by caspase-3, Z-DEVD-R110 is converted to a fluorescent compound whose fluorescent signal is proportional to the amount of caspase-3 activity in the cells. After adding the substrate Z-DEVD-R110, the plate is incubated for 1.0-1.5 hour, and the resulting fluorescence measured using a Victor^3^ microplate fluorometer (**PerkinElmer Life and Analytical Sciences**, Shelton, CT) at excitation and emission wavelengths of 485 nm and 535 nm, respectively.

**The annexin V assay**

Annexin V positive cells were quantified with an annexin V-FITC assay (Apoptosis Detection Kit, R&D Systems, Minneapolis, MN) and quantified using flow cytometry. Annexin V binds to phosphatidyl serine, which is translocated from the inner leaflet of the plasma membrane to the outer leaflet in the early stage of apoptosis.

**SUPPLEMENTARY FIGURE LENGENDS**

**Supplementary Figure 1** Sigma-2 ligands increased the percentage of annexin V and TUNEL positive cells. EMT-6 cells were either left untreated or treated with WC-26 (40 μM) for 48 h, SV119 (100 μM) for 16 h, or RHM-138 (40 μM) for 16 h; annexin V positive cells and TUNEL positive cells were quantified by flow cytometry. The data showed that the percentage of annexin V positive cells was increased from 7 ± 1.1% for the untreated cells to 96.6 ± 2.7%**,** 73.5 ± 2.4%, and 48 ± 6.4% after treatment with WC-26, SV119 and RHM-138, respectively. TUNEL positive cells increased from 4.57 ± 2.4% for the untreated cells to 48 ± 2.4%, 36 ± 3.0%, and 39.2 ± 3.5% for the cells treated with WC-26, SV119 and RHM-138, respectively. **p* < 0.0005 compared to the untreated control.

**Supplementary Figure 2** RHM-138 induced caspase 8 and 9 activation. MDA-MB435 cells were treated with 40 µM RHM-138 for 0, 1, 2, 4, 8, 16 and 24 h. Caspase 8 and 9 cleavage was analyzed by western blot. RHM-138 induced caspase 8 and 9 cleavage, suggesting that RHM-138 may trigger apoptosis via both the intrinsic and extrinsic pathways.
